# PDL1 Expression on Plasma and Dendritic Cells in Myeloma Bone Marrow Suggests Benefit of Targeted anti PD1-PDL1 Therapy

**DOI:** 10.1371/journal.pone.0139867

**Published:** 2015-10-07

**Authors:** Anne-Marit Sponaas, Neda Nejati Moharrami, Emadoldin Feyzi, Therese Standal, Even Holth Rustad, Anders Waage, Anders Sundan

**Affiliations:** 1 KG Jebsen Centre for Myeloma Research, Department of Cancer Research and Molecular Medicine, Norwegian University of Science and Technology, Trondheim, Norway; 2 CEMIR, Centre of Molecular Immune Regulation, Norwegian University of Science and Technology, Trondheim, Norway; 3 Department of Hematology, St.Olavs University Hospital, Trondheim, Norway; University Medical Center of the Johannes Gutenberg University of Mainz, GERMANY

## Abstract

In this study we set out to investigate whether anti PDL1 or PD–1 treatment targeting the immune system could be used against multiple myeloma. DCs are important in regulating T cell responses against tumors. We therefore determined PDL1 and PDL2 expression on DC populations in bone marrow of patients with plasma cell disorders using multicolour Flow Cytometry. We specifically looked at CD141^+^ and CD141^-^ myeloid and CD303^+^ plasmacytoid DC. The majority of plasma cells (PC) and DC subpopulations expressed PDL1, but the proportion of positive PDL1+ cells varied among patients. A correlation between the proportion of PDL1^+^ PC and CD141^+^ mDC was found, suggesting both cell types could down-regulate the anti-tumor T cell response.

## Introduction

Multiple myeloma is an incurable cancer and new therapies are urgently needed. In some advanced cancers encouraging results have been obtained after treating patients with anti PDL1 or PD–1 therapy. PD–1 (Programmed cell death–1, CD279) will, when present on T- and NK-cells, inhibit T-cell effector functions after engagement through its ligands PDL1 or PDL2 [1]. Thus, the rationale behind treatment with humanized anti PDL1 or PD–1 antibody therapy such as Nivolumab and Pidilizumab is to block this ligation in vivo [2]. Many tumors express PDL1. This molecule can constrain anti-cancer immune responses [1,3]. Although not detected on normal PC, myeloma cell lines and primary myeloma cells in the bone marrow up-regulate PDL1, and its ligand PD–1 is found on a proportion of T-cells in myeloma patients [4–7]. Although anti PD–1 treatment alone have so far not been effective [8], It is still important to characterize the expression of PDL1 and PDL2 on subsets of leukocytes to verify whether there are differences between myeloma patients. If this is the case, only subgroups of patients may respond to the therapy. In addition, we would like know whether responses to PD1 treatment could be restricted to patients with tumor cells expressing PDL1 [9].

Although PDL1^+^ tumors can suppress T and NK cell functions, other cells in the microenvironment may be equally important. Two papers describing expression of PDL1 on PC and pDC in myeloma were recently published [6,7]. However, the expression of PDL1 on subtypes of DC in tumors is so far largely undefined. DCs are superior to monocytes in controlling T-cell responses [10]. Four main populations of DCs have been identified in the blood and peripheral organs of humans [11]. Inflammatory DCs are similar to monocytes; they express CD11c and MHC class II, but not the myeloid DC marker CD1c. Their role in the regulation of T cell responses in humans is unclear and was therefore left out of the analysis. CD303^+^ plasmacytoid DC (pDC), are rapidly recruited to sites of inflammation where they produce cytokines and influence the local immune response [12]. CD1c^+^ myeloid DCs (mDC), which are most effective at controlling T cells, have two sub-types; CD1c^+^CD141^+^ DC (CD141^+^ DC), and CD1c^+^CD141^-^ DCs (CD141^-^ DC) [13,14]. The CD141^+^DCs control the CD8+ T cell responses and the CD141^-^DCs regulate the CD4+ T cell responses [11]. In this study we set out to determine the PDL1 and PDL2 expression on these 3 DC populations.

## Materials and Methods

### Bone marrow samples

Bone marrow cells and blood were collected in sodium heparin (Wockhardt,Wrexham, UK) from the iliac crest from patients suffering from multiple myeloma. The patients were registered in the Norwegian Myeloma Biobank and enrolled in the study. The study was approved by the Regional Ethics Committee (REK 2011–2029). The donors were classified as healthy, MGUS or multiple myeloma according to the International Myeloma Working Group (IMWG) criteria [15]. Patient’s data is shown in table in Supporting Information (S1 File). Samples were obtained after written consent. Bone marrow PC percentage was determined in May Grunwald Giemsa stained smears as part of standard diagnostic procedures.

### Antibodies

Anti-human CD141FITC (AD5-14H12), (CD3 (SK7), CD19 (SJ25C1), CD138 (MI15), CD56 (CMSSB), CD15 (VIM6C)) all PE conjugated, HLADRV450 (L234), CD45V500 (HI30), CD38PE-Cy7 (HIT2), CD19APCCy7 (SJ25C1) was obtained (BD Biosciences, Stockholm, Sweden). CD34PE (4H11), CD235aPE (HIR2), CD11cAlexa Flour 700 (B-ly6), CD1cPECy7 (L161), CD273APC (MH18), CD274APC (MIH1) and human FcReceptor binding inhibitor was obtained (eBioscience,San Diego,CA,USA), and CD303 PerCpPECy 5.5 (201A) were from BioLegend, San Diego,CA, USA. FACS lysing buffer was obtained from BD.

### Flow Cytometry

Un separated bone marrow cells were stained with a cocktail of antibodies against human CD141 lineage (CD3, CD19 CD15 CD56, CD138, CD34, and CD235a, CD45, HLADR, CD11c, CD303, CD1c and CD274 or CD273 for 30 min on ice after 20 min incubation with human Fc Receptor binding inhibitor. RBCs were lysed and cells fixed after staining. Flow cytometry was performed using LSR II (BD Biosciences, San Jose, CA, USA) with FACS Diva software (BD Biosciences). Samples were analysed with FlowJo 7.6 (TreeStar, Ashland, OR, USA). For the analysis of the mixed monocytes and DC populations, gates were set on live cells with forward and side scatter and doublets were gated out. Lineage and CD45^-^ were excluded and % PDL1 (CD274) or PDL2 (CD273) were determined on the gated CD11c^+^, HLA DR^+^ cells. Some of the patients also donated peripheral blood for this study.

Myeloma cells were stained in a separate panel with CD19, CD138, CD38, CD45 and CD274 antibodies. Gates were set on FSC and SSC and CD19^+^ cells were excluded. Myeloma cells were identified as CD19-CD38+ cells. There was a variation in the expression of CD138 and CD45 on the CD19-CD38+ myeloma cells amongst the patients.

### Statistical Analysis

Tests were performed using Graph Pad Prism 5 software. Correlations were determined using Spearman’s Test. Comparisons between groups were done with Mann Whitney. Significance was determined as p< 0.05. We chose to correlate % PDL1 expression with % plasma cell from the stained smears since bone marrow biopsy and ISS score were not available for all patients in this study.

## Results

### PDL1 expression in myeloma bone marrow

**Fig 1:**
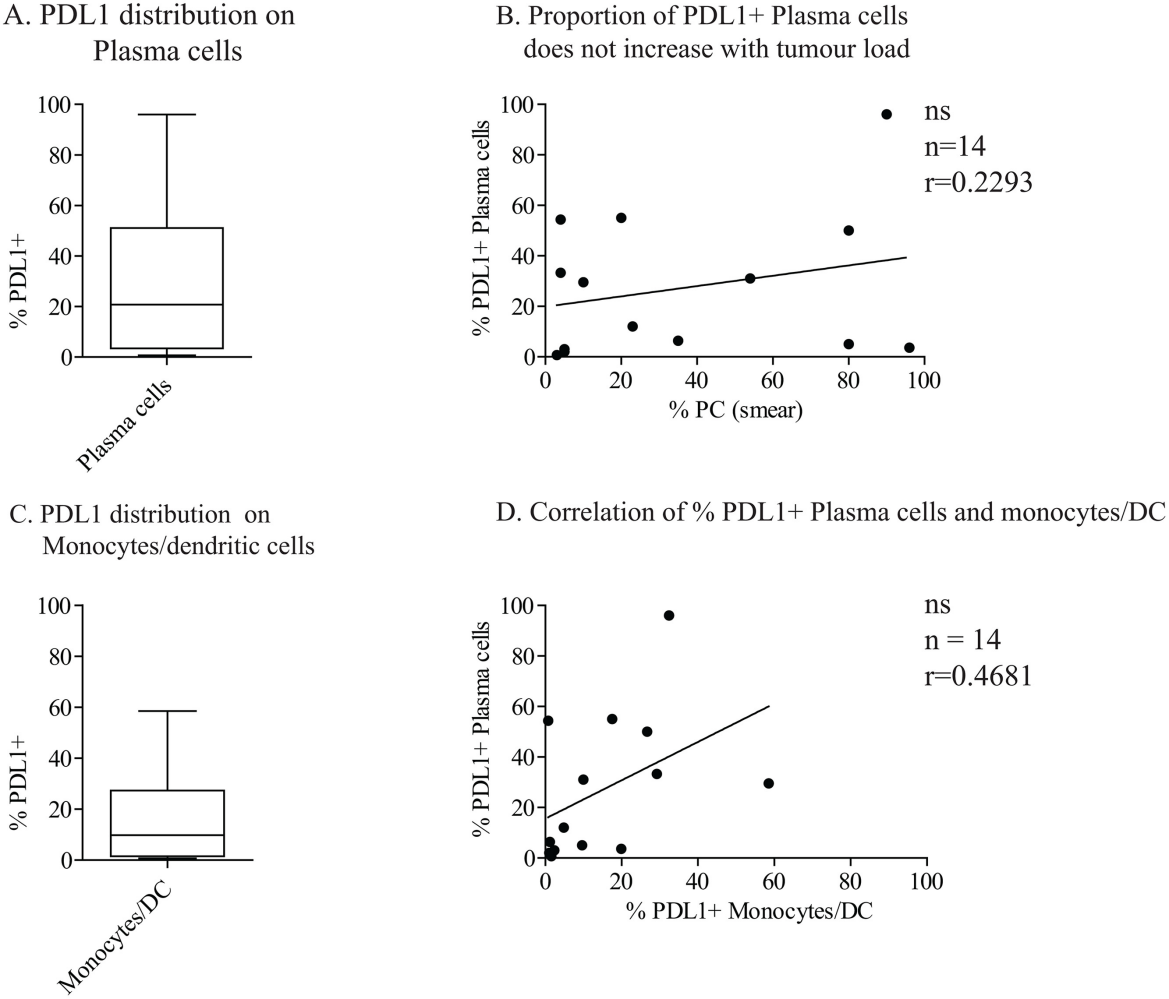
Expression of PDL1 on PC and monocytes in myeloma bone marrow. (A) PDL1 on plasma cells: Bone marrow cells were stained with antibodies against CD45, CD138, CD38, CD19, and CD274 (PDL1). Gates were set on FSC and SSC and doublets and CD19+ cells were excluded. Gating strategy is shown in Fig A in S2 File. The distribution of % PDL1^+^ PC in the bone marrow of patients (n = 14) is shown. (B) Proportion of PDL1^+^ PC does not increase with tumor load. The % PDL1^+^ gated CD38^+^CD19^-^ PC versus % bone marrow plasma cells is plotted. Each dot represents one patient. P values were calculated from a Spearman’s test (n = 14). (C) PDL1 on monocytes and DCs: Bone marrow cells were stained with antibodies against lineage (CD3, CD19, CD56, CD138, CD15, CD34, and CD235a), CD45, HLADR, and CD11c. The gating strategy is shown in Supplementary S1B Fig. Gates were set on FSC and SSC, doublets excluded, and gates further set on lineage- CD45^+^cells. Figure shows distribution of % PDL1+ monocytes/DC in the bone marrow of patients (n = 14). (D) Correlation of % PDL1+ PC and monocytes/DC; % PDL1^+^CD11c^+^DR^+^ monocytes/DC versus % PDL1^+^CD38^+^CD19^-^ plasma cells is plotted. Each dot represents one patient. P value was calculated from a Spearman’s test.

In this study we used flow cytometry to determine the expression of PDL1 and PDL2 on unseparated bone marrow and blood cells from patients with multiple myeloma. We chose to use unseparated bone marrow and blood samples because cell isolation procedures may affect surface expression of the markers. First, we determined the fractions of PDL1^+^ and PDL2^+^ PC and DC and monocytes in bone marrow aspirates. For myeloma cells, bone marrow cells were stained with anti CD45, CD19, CD38 and CD138, as well as PDL1 (CD274), or PDL2 (CD273), as described in Material and Methods and in supplementary information (Fig A in S2 File) PCs were identified as CD19^-^, CD38^high^ cells since staining with anti-CD45 and CD138 varied (data not shown). Patients with more than 5% PDL1^+^ PCs were defined as PDL1 positive [2]. Ten out of fourteen patients (71%) had PDL1^+^ myeloma cells. There was, however, variation in the abundance of PDL1^+^ PCs among the patients, ranging from 5% to 96% positive PCs (Fig 1A). The proportion of PDL1^+^ PCs did not increase with tumor load (Fig 1B), suggesting that PDL1 expression is not determined directly by the tumor burden, but rather that it is the bone marrow microenvironment that regulates PDL1 expression.

Next we measured the proportion of PDL1 and PDL2 expressing monocytes/DCs. As both monocytes and DCs express MHC class II and CD11c [11], cells were stained with a cocktail of antibodies against lineage cells (CD138, CD56, CD34, CD235a, CD19, CD15 and CD3), CD45, HLADR, CD11c and PDL1 (CD274) or PDL2 (CD273). Briefly, gates were set on live cells, CD45^+^ and lineage negative cells and a dot plot of CD11c and HLADR staining of such gated cells is shown (Fig B in S2 File). The majority of patients -71% (10/14) were PDL1^+^; i.e. had more than 5% PDL1^+^ cells. The proportion of PDL1^+^ monocytes/DCs varied among patients from 6% to 58% (Fig 1C). Most patients with high a proportion of PDL1^+^ PC also had a high proportion of PDL1^+^ monocytes/DCs (Fig 1D), although there was no significant correlation between high expression on PCs and monocytes/DCs. Interestingly, two patients with no PDL1 on their PCs had PDL1^+^ (>5%) monocytes/DCs.

The blood monocytes/DCs from healthy donors expressed no PDL1 (data not shown). Furthermore, no PDL2 positive PCs or monocytes /DCs were detected (Figs C and E in S2 File and data not shown), suggesting that PDL2 expression is not important for attenuating T-cell responses in myeloma bone marrow.

### PDL1 expression on DC subtypes in myeloma bone marrow

**Fig 2:**
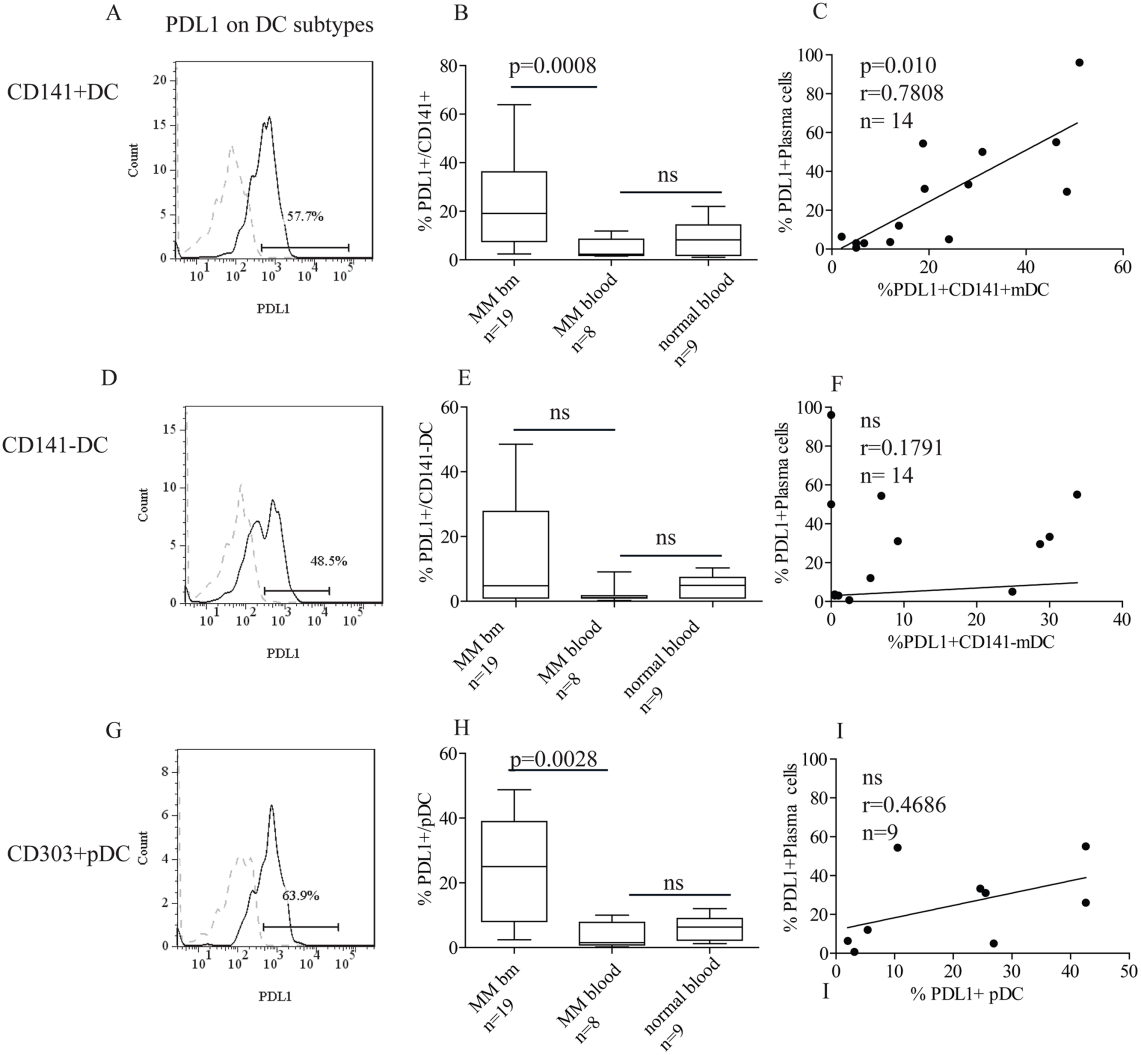
DC subtypes express PDL1 in myeloma bone marrow. Bone marrow and blood were stained with antibodies against CD141, lineage (CD3, CD19, CD56, CD138, CD15, CD34, and CD235a), CD45, HLADR, CD303, CD1c, and CD11c. The gating strategy is shown in Fig D in S2 File). Three DC populations were analysed; CD141^+^ (CD141^+^DC) (panels A-C), CD141^-^ (CD141^-^DC) (panels D-F), and CD303^+^DC (pDC) (panels G-I). PDL1 staining on one representative patient (panels A, D, G). Fluorescence minus one (FMO), (dotted line), was used as negative control and the percentage indicates PDL1^+^ cells of the gated DC population. Panels B, E, and H show percentage of PDL1^+^ cells within the (B) CD141^+^ DC, (E) CD141^-^ DC and (H) CD303^+^ pDC populations in the bone marrow (n = 19), blood (n = 8) from patients, or blood from age matched (median age 61) healthy controls (n = 9). (median age of patients 61). Statistical analysis was performed with Mann Whitney Test. Panels C, F, and I show concomitant expression levels on bone marrow DC subtypes and plasma cells in individual patients. Each dot represents one patient. P values were calculated from Spearman’s tests.

In line with earlier reports, we found PDL1 expression on pDCs [7]. Thus, 81% (13/16) of patients had more than 5% positive pDC (Fig 2). Interestingly, high proportions of PDL1^+^CD141^+^DCs were detected in the patients’ bone marrow (Fig 2B–2C and 2H–2I). Thus, the majority, 81% (17/21) had PDL1^+^CD141^+^ DC. In contrast, only 48% (10/21) of the patients had PDL1^+^ CD141^-^ DCs. This suggested that PDL1 expression on CD141^+^ DCs could attenuate the activity of tumor reactive T-cells.

Again, there were variations in the proportion of PDL1^+^ DCs among the patients (Fig 2B, 2E and 2H). Furthermore, we found no significant correlation between % PC (i.e. tumor load), and proportion of any of the PDL1^+^ DC populations (Fig F in S2 File). PDL1 is known to be up-regulated on DCs in response to inflammatory stimuli [16,17]. We also found that patients with high proportions of PDL1^+^ PCs had many PDL1^+^ CD141^+^ DCs (Fig 2C), suggesting that agents responsible for the PDL1 up-regulation were similar for both cell populations.

The PDL1^+^ DCs were confined to the bone marrow (Fig 2), as only a small fraction of the myeloma patients had PDL1^+^ DCs in the blood (2/8 patients had PDL1^+^ blood CD141^+^ DCs, 1/8 had PDL1^+^ CD141^-^ DCs), indicating that the PDL1^+^ DCs accumulate in the organ affected by malignant cells.

## Discussion

Since dendritic cells (DC) are important in regulating T cell responses, we determined PDL1 and PDL2 expression on CD141^+^ and CD141^-^ myeloid (mDCs) and CD303^+^ plasmacytoid dendritic cells (pDCs), as well as on plasma cells (PC) in the blood and bone marrow in groups of patients with MGUS (monoclonal gammopathy of undetermined significance) and multiple myeloma. None of the patients expressed PDL2, but PDL1 was found on the majority of PC and DC subpopulations in the bone marrow. The proportion of PDL1^+^ cells varied among patients. Interestingly, there was a positive correlation between the proportion of PDL1^+^ PC and CD141^+^ mDC suggesting that both cell types could inhibit the anti-tumor T cell response. It was shown previously that PDL1 expression on pDCs was important for regulation of T cell responses in myeloma patients [7]. We found that CD141^+^DCs also expressed PDL1, which is important as these cells control the tumor-specific cytotoxic T cell responses. We propose that myeloma patients may benefit from anti PD–1/PDL1 treatment. However, it may be necessary to investigate PDL1 expression on dendritic cells in addition to plasma cells to identify patients who may benefit from the treatment. The myeloma patients expressed PDL1 in bone marrow but not in peripheral blood, suggesting that factors present in the tumor environment is important for PDL1 expression. The expression of PDL1 on DCs and myeloma cells may be equally important in attenuating anti tumor immunity. It is not known if there is a threshold of PDL1 expression in the tumor or its microenvironment for attenuating anti-tumor activity. At least 6 clinical studies targeting PDL1 or PD–1 in multiple myeloma have recently been launched (www.clinicaltrials.gov/). Some results were published from one of these studies (reported at ASH 2014). Although no objective response was observed in this study, 67% of the patients had stable disease. Further analysis of the PDL1 expression as shown here may be beneficial in evaluating the response to .PD1 treatment in multiple myeloma.

## Supporting Information

S1 File Patient information. The patients were classified according to the International Myeloma Working Group (IMWG) criteria. Where limited sample volume was available only part of the analysis was performed.
ND: not done, NA: not applicable
(DOCX)

S2 File Gating strategy of plasma cells and dendritic cells. (A) CD38 and CD138 expression on plasma cells: Unseparated bone marrow cells were stained with a panel of antibodies against CD45, CD138, CD38, CD19, and CD274 (PDL1) before analysis. Gates were set on FSC and SSC and doublets and CD19+ cells were excluded. Dot plot of CD138 and CD38 staining of a patient who expressed CD138 is shown. Histogram shows PDL1 expression of the gated cells. FMO (dotted line) was used as negative control and the percentage indicates PDL1+ cells of the gated population. (B) Staining of monocytes/DC: Unseparated bone marrow cells were stained with a cocktail of antibodies against, lineage (CD3, CD19, CD56, CD138, CD15, CD34, and CD235a), CD45, HLADR. and CD274 (PDL1) or CD273(PDL2). Briefly, gates were set on FSC and SSC, doublets were excluded, and gates were further set on lineage- CD45^+^cells (left panel) and a gate was then set on CD11c and HLA DR double positive cells (right hand panel). Histogram shows PDL1 expression of the gated cells. FMO (dotted line) was used as negative control and the percentage indicates PDL1+ cells of the gated population. (C) Histogram of PDL2 expression on CD45^-^CD38^+^ bone marrow plasma cells. FMO (dotted line) was used as negative control and the percentage indicates PDL1^+^ cells of the gated population. (D) Gating strategy for subgroups of DCs: Unseparated blood and bone marrow cells as indicated were stained with a cocktail of antibodies against CD141, lineage (CD3, CD19, CD56, CD138, CD15, CD34, and CD235a), CD45, HLADR, CD303, CD1c, and CD11c and CD274 (PDL1) and analysed on an LSR II Flow cytometer. Briefly, gates were set on FSC and SSC, doublets were excluded, and gates were further set on lineage^-^ CD45^+^cells, and a gate was then set on CD11c and HLA DR double positive cells, before the CD1c and CD141, and the CD1c and CD303 profiles are shown. (E) i. PDL2 expression on monocytes/dendritic cells. Histogram of PDL2 (CD273) staining of CD11c^+^ HLA DR^+^ gated monocyte/DC population in the bone marrow of a representative patient. The dotted line shows the negative control as in S1C Fig. ii. Histogram of in vitro cultured DC from PBMC. Monocytes were isolated from PBMC by adherence and cultured with 40 ng/ml GM-CSF (R&D) and 100ng/ml IL4 (R&D) for 7 days before adding 10ng/ml LPS (InVivogen) for 4 hrs. The dotted line shows isotype control. (F) Proportions of PDL1^+^ DCs do not increase with tumor load. These figures show the percent PDL1^+^ versus % PC (May Grunwald Giemsa stain) of all 3 DC populations. Each dot represents a patient. P value was calculated from a Spearman’s test.
(PDF)
