# Supplementary material for: PDL1 Expression on Plasma and Dendritic Cells in Myeloma Bone Marrow Suggests Benefit of Targeted anti PD1-PDL1 Therapy
Source: PLoS One. 2015 Oct 7;10(10):e0139867. doi: 10.1371/journal.pone.0139867 (PMC4596870; doi:10.1371/journal.pone.0139867)
Supplement: S1 File — The patients were classified according to the International Myeloma Working Group (IMWG) criteria. Where limited sample volume was available only part of the analysis was performed. ND: not done, NA: not applicable (DOCX) [file pone.0139867.s001.docx]

S1 File. Patient information:

| patient | Sex | Age | % PC (smear) | M comp/light chain (g/L) | subtype | Treatment | ISS | Diagnosis |
| --- | --- | --- | --- | --- | --- | --- | --- | --- |
| 1 | f | 51 | 90 | 25,7 | IgG κ | new diagnose/ untreated | 1 | symptomatic myeloma |
| 2 | m | 67 | 80 | 24.1 | IgG λ | new diagnose/ untreated | 2 | symptomatic myeloma |
| 3 | f | 51 | <5 | 1 | IgG λ | relapse/treated | 2 | symptomatic myeloma |
| 4 | m | 37 | 56 | 92 | light chain λ | new diagnose/ untreated | 3 | symptomatic myeloma |
| 5 | f | 63 | 96 | - | non-secretory | relapse/ not receiving treatment | 3 | symptomatic myeloma |
| 6 | m | 72 | 80 | 48,4 | IgG κ | relapse/not receiving treatment | 2 | symptomatic myeloma |
| 7 | m | 56 | 25 | 49.2 | IgG λ | new diagnose/un treated | 2 | symptomatic myeloma |
| 8 | m | 43 | 6 | 0.4 | light chain λ | NA | NA | MGUS |
| 11 | m | 62 | 35 | 16,8 | light chain κ | new diagnose/ untreated | 2 | symptomatic myeloma |
| 12 | f | 53 | 20 | 47 | IgA κ | relapse/treated | 1 | symptomatic myeloma |
| 14 | m | 74 | 5 | NA | NA | NA | NA | no plasma cell disease |
| 16 | m | 49 | 9 | 43,1 | IgG κ | relapse/no treatment | 3 | symptomatic myeloma |
| 17 | f | 60 | 10 | 13 | IgA κ | new diagnosed, untreated | ND | symptomatic myeloma |
| 18 | f | 86 | 3 | - | - | - | - | normal |
| 19 | f | 70 | 95 | 6,1 | light chain κ | new diagnose/un treated | 3 | multiple myeloma/ primary plasma cell leukemia |
| 20 | f | 72 | 20 | 32 | IgG κ | new diagnose/un treated | ND | myeloma |
| 21 | f | 77 | 11 | 50 | IgG κ | new diagnose/un treated | 3 | symptomatic myeloma |
| 22 | f | 63 | 2 | 0,1 | IgG κ | MGUS, untreated | NA | MGUS |
| 23 | f | 79 | 25 | 41 | IgG κ | new diagnose/un treated | 3 | symptomatic myeloma |
| 24 | m | 88 | 23 | 23 | IgG κ | relapse, no treatment | 3 | symptomatic myeloma |
| 25 | f | 46 | 35 | ND | ND | ND | ND | myeloma |
| 26 | f | 70 | 3 | ND | IgG κ | relapse, no treatment | 2 | symptomatic myeloma |
| 27 | m | 65 | 5 | 29.2 | IgG κ | relapse, currently treated | 3 | symptomatic myeloma |
| 28 | m | 61 | 58 | ND | ND | ND | ND | myeloma |
| 29 | m | 57 | 4 | 16,8 | light chain κ | new diagnose, un treated | 3 | symptomatic myeloma |

Legend

The patients were classified according to the International Myeloma Working Group (IMWG) criteria. Where limited sample volume was available only part of the analysis was performed.

ND: not done, NA: not applicable
