# Supplementary material for: PDL1 Expression on Plasma and Dendritic Cells in Myeloma Bone Marrow Suggests Benefit of Targeted anti PD1-PDL1 Therapy
Source: PLoS One. 2015 Oct 7;10(10):e0139867. doi: 10.1371/journal.pone.0139867 (PMC4596870; doi:10.1371/journal.pone.0139867)

S1 Fig

Gating strategy:

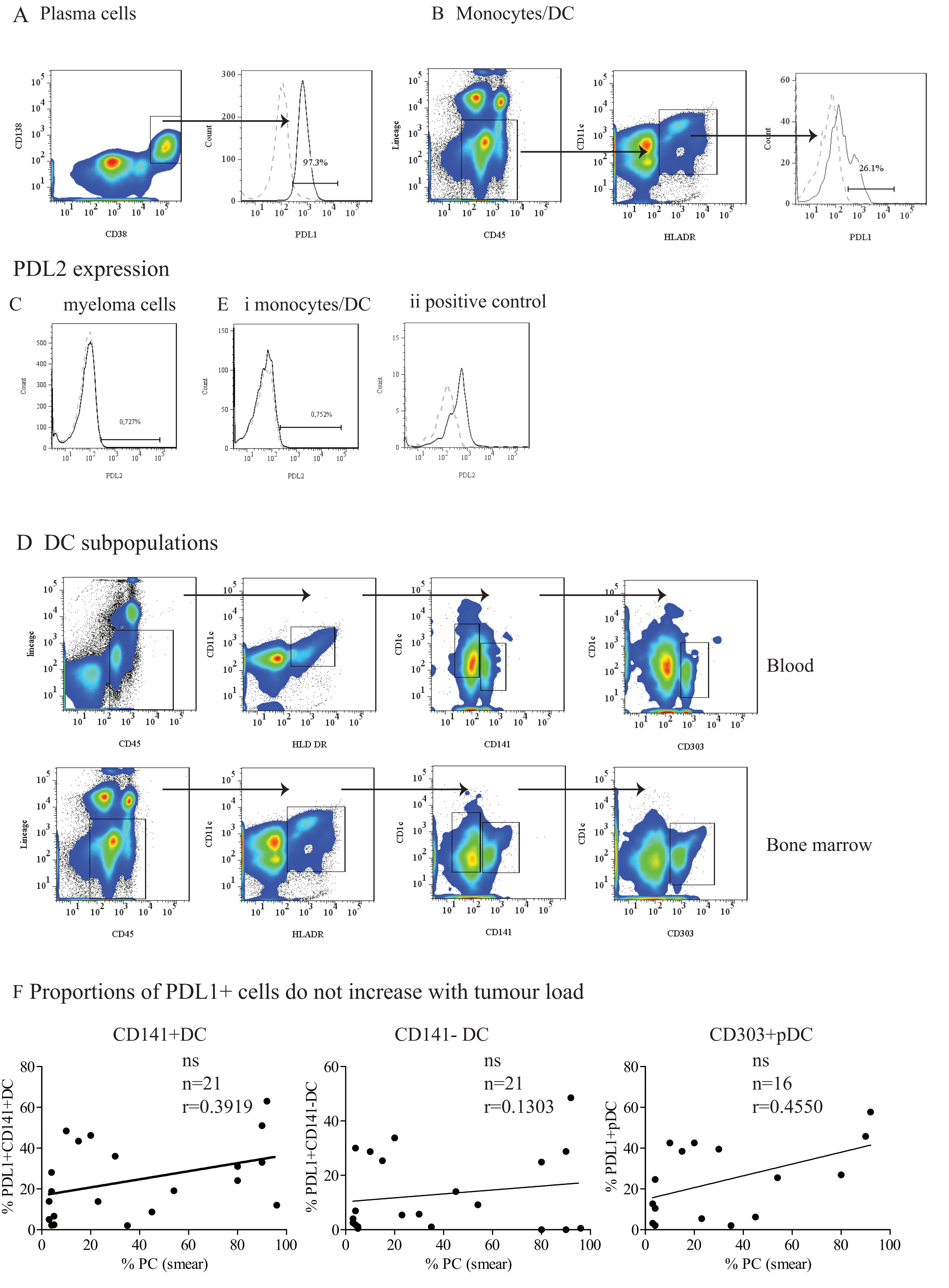

Supplement: S2 File — (A) CD38 and CD138 expression on plasma cells: Unseparated bone marrow cells were stained with a panel of antibodies against CD45, CD138, CD38, CD19, and CD274 (PDL1) before analysis. Gates were set on FSC and SSC and doublets and CD19+ cells were excluded. Dot plot of CD138 and CD38 staining of a patient who expressed CD138 is shown. Histogram shows PDL1 expression of the gated cells. FMO (dotted line) was used as negative control and the percentage indicates PDL1+ cells of the gated population. (B) Staining of monocytes/DC: Unseparated bone marrow cells were stained with a cocktail of antibodies against, lineage (CD3, CD19, CD56, CD138, CD15, CD34, and CD235a), CD45, HLADR. and CD274 (PDL1) or CD273(PDL2). Briefly, gates were set on FSC and SSC, doublets were excluded, and gates were further set on lineage- CD45+cells (left panel) and a gate was then set on CD11c and HLA DR double positive cells (right hand panel). Histogram shows PDL1 expression of the gated cells. FMO (dotted line) was used as negative control and the percentage indicates PDL1+ cells of the gated population. (C) Histogram of PDL2 expression on CD45-CD38+ bone marrow plasma cells. FMO (dotted line) was used as negative control and the percentage indicates PDL1+ cells of the gated population. (D) Gating strategy for subgroups of DCs: Unseparated blood and bone marrow cells as indicated were stained with a cocktail of antibodies against CD141, lineage (CD3, CD19, CD56, CD138, CD15, CD34, and CD235a), CD45, HLADR, CD303, CD1c, and CD11c and CD274 (PDL1) and analysed on an LSR II Flow cytometer. Briefly, gates were set on FSC and SSC, doublets were excluded, and gates were further set on lineage- CD45+cells, and a gate was then set on CD11c and HLA DR double positive cells, before the CD1c and CD141, and the CD1c and CD303 profiles are shown. (E) i. PDL2 expression on monocytes/dendritic cells. Histogram of PDL2 (CD273) staining of CD11c+ HLA DR+ gated monocyte/DC population in the bone marrow of a r [file pone.0139867.s002.pdf]
